# A Question—Protrusion

**Published:** 1859-10

**Authors:** 


### A QUESTION—PROTRUSION.

Why is it that gold fillings especially on the approximal portions of the front teeth, after they have been worn a few years, in many cases protrude from the cavity, while the filling is perfect and solid in the cavity? What should be done in such a case?

Q.

A solution of the above query is found most probably in the fact of the wearing away of the dentine and enamel that forms the border of the cavity.

The filling in such a case should be dressed down smooth with the present border of the cavity and finished as usual. There is no danger of displacing such plugs by dressing. T.

